# Multi-class boosting for the analysis of multiple incomplete views on microbiome data

**DOI:** 10.1186/s12859-024-05767-w

**Published:** 2024-05-14

**Authors:** Andrea Simeon, Miloš Radovanović, Tatjana Lončar-Turukalo, Michelangelo Ceci, Sanja Brdar, Gianvito Pio

**Affiliations:** 1 BioSense Institute, University of Novi Sad, dr Zorana Djindjića 1, Novi Sad, 21000 Serbia; 2 Faculty of Sciences, University of Novi Sad, Trg Dositeja Obradovića 3, Novi Sad, 21000 Serbia; 3 Faculty of Technical Sciences, University of Novi Sad, Trg Dositeja Obradovića 6, Novi Sad, 21000 Serbia; 4 Department of Computer Science, University Bari Aldo Moro, Via Orabona 4, 70125 Bari, Italy; 5 Big Data Laboratory, National Interuniversity Consortium for Informatics (CINI), Via Ariosto 25, 00185 Rome, Italy; 6 Department of Knowledge Technologies, Jožef Stefan Institute, Jamova cesta 39, 1000 Ljubljana, Slovenia

**Keywords:** Multi-view learning, Incomplete views, Multi-armed bandits, Boosting, Microbiome analysis

## Abstract

**Background:**

Microbiome dysbiosis has recently been associated with different diseases and disorders. In this context, machine learning (ML) approaches can be useful either to identify new patterns or learn predictive models. However, data to be fed to ML methods can be subject to different sampling, sequencing and preprocessing techniques. Each different choice in the pipeline can lead to a different view (i.e., feature set) of the same individuals, that classical (single-view) ML approaches may fail to simultaneously consider. Moreover, some views may be incomplete, i.e., some individuals may be missing in some views, possibly due to the absence of some measurements or to the fact that some features are not available/applicable for all the individuals. Multi-view learning methods can represent a possible solution to consider multiple feature sets for the same individuals, but most existing multi-view learning methods are limited to binary classification tasks or cannot work with incomplete views.

**Results:**

We propose irBoost.SH, an extension of the multi-view boosting algorithm rBoost.SH, based on multi-armed bandits. irBoost.SH solves multi-class classification tasks and can analyze incomplete views. At each iteration, it identifies one winning view using adversarial multi-armed bandits and uses its predictions to update a shared instance weight distribution in a learning process based on boosting. In our experiments, performed on 5 multi-view microbiome datasets, the model learned by irBoost.SH always outperforms the best model learned from a single view, its closest competitor rBoost.SH, and the model learned by a multi-view approach based on feature concatenation, reaching an improvement of 11.8% of the F1-score in the prediction of the Autism Spectrum disorder and of 114% in the prediction of the Colorectal Cancer disease.

**Conclusions:**

The proposed method irBoost.SH exhibited outstanding performances in our experiments, also compared to competitor approaches. The obtained results confirm that irBoost.SH can fruitfully be adopted for the analysis of microbiome data, due to its capability to simultaneously exploit multiple feature sets obtained through different sequencing and preprocessing pipelines.

**Supplementary information:**

The online version contains supplementary material available at 10.1186/s12859-024-05767-w.

## Introduction

Over the last few years, microbiome dysbiosis has been associated with many diseases, since it tends to occur as an accompanying symptom with a higher prevalence than usual in disease conditions. Microbiome alterations can be related to some types of cancer [1–3] or, through the so-called *microbiota-gut-brain* axis, to neurodevelopmental conditions such as the Autism Spectrum Disorder (ASD) [4, 5].

Sequencing of microbiome samples is considered convenient, especially when other tests are impossible, hard or invasive to perform. However, despite the reduction of sequencing costs, they can still be problematic, specifically for large microbiome studies or whole genome studies. In this context, the Next Generation Sequencing (NGS) technology has revolutionized microbiome sequence analysis. Indeed, while initially the research targeted only hypervariable regions of conserved genes such as 16S ribosomal RNA (rRNA) gene, more recently, it has been expanded on longer reads [6, 7] and on the whole genome. In 16S (*amplicon*) metagenomic sequencing, short chains (< 500 bp) of nucleotides from 16S rRNA gene are amplified by PCR and then read out. This is further used to identify and differentiate multiple microbial species from multiple samples at once. In spite of simplification, this approach suffers from several drawbacks: poor resolution (only up to the level of species); poor diversity detection; sample source dependency, specifically on sampling, sequencing and analysis protocols, such DNA extraction kits and alignment algorithms [6–8]; poor reproducibility [6].

The sequencing of the whole genome solved some of the above-mentioned limitations, among which the most important is identification accuracy. This sequencing technique, called *shotgun*, involves randomly breaking up the genome into small overlapping DNA fragments, that are sequenced individually and virtually reassembled. Even if more precise, it also exhibits some drawbacks: the issue about the sample source dependency is not solved, and it is generally more expensive.

16S amplicon sequencing was initially very popular, and the amount of available data collected with this technique is currently much larger than that of the shotgun counterpart. However, several studies now aim to adopt both approaches [4], even if their simultaneous exploitation in a pipeline of analysis is challenging.

In the literature, we can find several attempts involving the adoption of machine learning (ML) techniques for the analysis of microbiome data [9–11]. However, to the best of the authors’ knowledge, there is no work aiming to consider simultaneously 16S and shotgun data in the construction of ML models. A simple solution would consist in the concatenation of the features representing the same individual, obtained through 16S and shotgun techniques. However, this approach can exacerbate the curse of dimensionality [12], which is already present in microbiome data due to the high unbalancing between the (usually low) number of individuals and the (usually high) number of features. In this context, *multi-view learning* approaches [12–14] represent a possible solution, since they mainly follow a *decision level* fusion, based on average/voting approaches, similarly to ensemble techniques. In these methods, different feature sets of the same instances (e.g., pictures of the same objects taken from different perspectives, features representing conceptually different aspects, or features obtained after the application of different preprocessing techniques) are called *views*, and a ML model is learned on each view separately. Subsequently, their output is combined and iteratively exploited to boost the performance of the learned models.

**Fig. 1:**
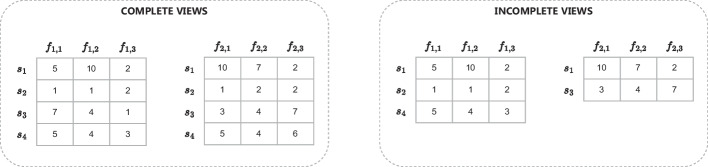
An example showing the difference between complete (on the left) and incomplete (on the right) views: rows correspond to the instances (individuals, in the case of microbiome studies); each view represents a different viewpoint of the instances (e.g., 16S and shotgun data, or features obtained through different preprocessing pipelines). In the case of incomplete views, we can observe that not all instances are represented according to both views (e.g., $s_3$ is not represented in the first view, while $s_2$ and $s_4$ are not represented in the second view

Among the pioneering techniques, we can mention the co-training framework [15], that works on two views in the semi-supervised setting. In this framework, unlabeled examples are labeled by each of the two classifiers, and the most reliable predictions are fed as new training examples to the classifier working on the other view, for the subsequent iteration(s). This approach was originally limited to working on exactly two views. Different variants have been proposed in the literature, such as the co-regularization technique [16, 17], that was mainly adopted for reducing overfitting issues. In [16], the authors extended it to work with more than two views, while in the [18], the authors proposed a multi-view learning approach able to work in the positive-unlabeled learning setting for gene network reconstruction.

In the work [19], the authors extended the well-known boosting algorithm Adaboost [20] to the multi-view learning setting. In [21], the authors proposed an efficient version using adversarial multi-armed bandits for selecting one of the views based on its usefulness, instead of repeatedly considering all views. Other approaches for multi-view learning exploit kernel-based algorithms. Relevant examples include the system proposed in [22], which uses within- and between-view regularization, and the work [23], where the authors formulate the learning problem on each view as a Multiple Kernel Learning (MKL) problem. Both approaches were compared with four different methods proposed in [12], along with the original Adaboost on concatenated views, and the system Mumbo [24], a multi-view algorithm for multi-class classification that exploits a separate instance weight distribution for each view. Among the considered competitors and the variants proposed in [12], rBoost.SH, which is based on partial information games, obtained the best results.

One of the strongest limitations of the aforementioned approaches is that they can only work when samples are fully observed on all the considered views. However, in real-world situations, some of the available observations may not be represented by all the views (i.e., they are partially observed). A relevant example in the context of microbiome data can be represented by individuals for whom we have a 16S sample but not a shotgun sample, or vice versa. In this scenario, the above-mentioned approaches cannot be adopted without a preprocessing step aimed to fill missing values, possibly introducing excessive approximations in the data. In [25], the authors proposed a multi-view model to complete missing values using a SOR-like optimization algorithm. The issue about incomplete views in multi-view learning has also been tackled in two recent papers: [14] considered data incompleteness for the multi-view clustering task, while [26] adopted a graph-based learning approach for classification with incomplete views.

Another limitation of most of multi-view methods comes from their inability to solve multi-class classification tasks. While some approaches like Mumbo [24] tackled this issue, to the best of the authors’ knowledge, none of them can simultaneously solve multi-class classification tasks and work on incomplete views.

In this paper, we aim to fill this gap by proposing a novel multi-view approach that is able to work on incomplete views (see an example in Fig. 1) to solve multi-class classification problems. We adopt the proposed method in the context of microbiome studies for two different scenarios: (i) to classify individuals according to 16S and/or shotgun microbiome data, which represent two, possibly incomplete, different views; (ii) to classify individuals considering multiple preprocessing pipelines to generate different views from the same 16S data. Through the former scenario we demonstrate the ability of our approach to fully exploit both views of the microbiome data, while in the latter we show the applicability of our approach to solve one of the critical issues of the analysis of microbiome data, namely, the selection of the best preprocessing pipeline(s), which in our case is fully automated.

The remainder of the paper is structured as follows: in ‘Methods’ section we describe the proposed approach in detail, starting from the background concepts it is based on; ‘Experimental setting’ section presents the considered tasks, together with the adopted datasets and the experimental setting considered; in ‘Results and discussion’ section we show and discuss the obtained results. Finally, in ‘Conclusion’ section we conclude the paper and present some avenues for future work.

## Methods

The approach we propose in this paper is based on the method rBoost.SH [12]. Specifically, we significantly extend it to make it able to solve multi-class classification tasks and to work with incomplete views. We call our approach irBoost.SH. In ‘Boost.SH and rBoost.SH: binary classifier via multi-view boosting from complete views’ section, we first briefly describe the methods Boost.SH and rBoost.SH, together with the main concepts they rely on. Then, in ‘The proposed method irBoost.SH: multi-class classifier via multi-view boosting from incomplete views’ section we provide the details about our method irBoost.SH.

### Boost.SH and rBoost.SH: binary classifier via multi-view boosting from complete views

In this subsection, we provide a quick overview of *Boost.SH*, which is the simplest version among the multi-view algorithms proposed in [12, 27], and then we describe its extension *rBoost.SH* which is based on adversarial multi-armed bandits. Both *Boost.SH* and *rBoost.SH* adopt a single instance weight distribution that is shared among the views, unlike the multi-armed version of AdaBoost [21], which allows each view to adopt its own weight distribution. We remind that the approach we propose in this paper is based on *rBoost.SH*, which can originally solve only binary classification tasks from complete views.

**Boost.SH** starts from training data $S = \{({\textbf{x}}_i, y_i)\}_{i=1}^{n}$, where ${\textbf{x}}_i = \{x_i^1, x_i^2,..., x_i^K\}$ is a training instance, $x_i^j \in {\mathbb {R}}^{q_j}$ is the instance $x_i$ represented according to the *j*-th view in its $q_j$-dimensional feature space, and $y_i \in {\mathcal {Y}}=\{-1, +1\}$ is the label of the instance $x_i$. Instances $({\textbf{x}}_i, y_i)$ are assumed to be independently and identically distributed according to a probability distribution *D* over ${\mathcal {X}} \times {\mathcal {Y}}$, where ${\mathcal {X}} \subseteq {\mathbb {R}}^q$ and $q=\sum _{j=1}^{K}q_j$. At the beginning, the shared instance weights are uniformly distributed.

For each iteration of the algorithm and for each view, a *weak classifier* is trained and, on the basis of the predictions on training data, an *edge* is computed, that somehow represents the current predictive accuracy of such a view. Afterwards, the algorithm selects the view with the largest edge as the winning view. The edge of the winning view and the corresponding classifier are used to update the shared instance weights for the subsequent iterations. Finally, after *T* iterations, *Boost.SH* builds a combined classifier as a weighted sum of the winning classifiers over all the *T* iterations.

Despite optimizing the *consistency* through the shared weight distribution, Boost.SH does not promote *diversity* [12]. In general, the algorithm is limited to learn only from the views with the maximal edge. The extreme situation happens when a view wins for all the iterations, that would degenerate to learning from one single view, discarding the possible contribution provided by the other(s).

This limitation has been overcome by its randomized version, called **rBoost.SH**, that is formalized in Algorithm 1. It incorporates *adversarial multi-armed bandits* (AMB) into a boosting process by computing probabilities ${\textbf{p}}_{1 \times K}$ for choosing the actions to take (i.e., the views to select as winner).

Auer et al. [28, 29] proposed different versions of AMB. The one of interest here is a partial information game algorithm, called EXP3.P, in which a *player* (an algorithm) and an *adversary* (views) compete, and the reward is the only information propagated to the player. Unlike other problems based on bandits, in this case, no statistical assumptions are made about the process followed to generate the rewards (stochastic bandits, for instance, use the assumption that rewards are generated from a given, pre-determined, distribution). In **rBoost.SH**, the AMB problem is addressed using the exponentially weighted average (EWA) forecaster algorithm [12, 30]. The forecaster algorithm updates the probability distribution over actions/views, such that the probability of choosing an action exponentially depends on the average rewards associated with it [12]. The update of this probability distribution is controlled by two parameters, namely $\sigma$ and $\gamma$.

Contrary to Boost.SH, rBoost.SH computes the predictions only along the selected view *j* (see line 5 in Algorithm 1). The reward for such a view is divided by the probability of selecting it (see line 9 in Algorithm 1), which encourages the views with low probability to be selected, promoting diversity.

**Algorithm 1:**
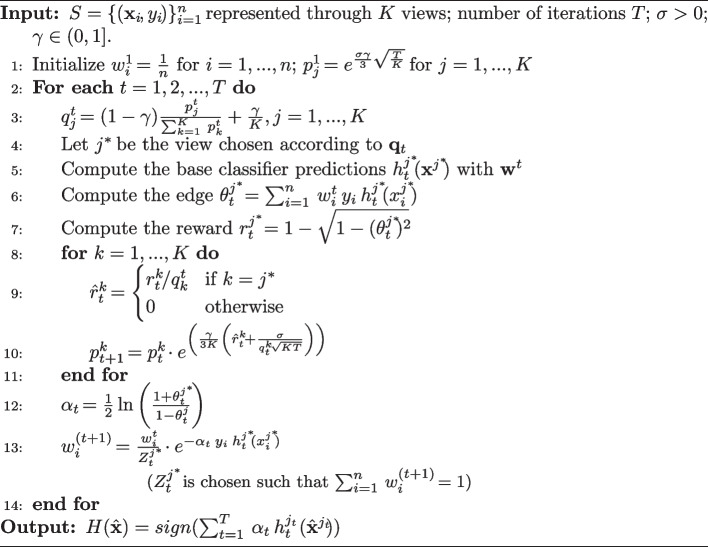
The method rBoost.SH

### The proposed method irBoost.SH: multi-class classifier via multi-view boosting from incomplete views

In this subsection, we present our method irBoost.SH, which overcomes the main limitations of rBoost.SH, namely, it can solve multi-class classification tasks and can also work with incomplete views, i.e., when some instances are not represented according to all the views.

The proposed method irBoost.SH is formalized in Algorithm 2. In this case, the set *S* consists of views of possibly different shapes: each instance can be present in one or more views, but not necessarily in all the views, and the number of features in each view can be different.

For each view *j*, with $j=1, 2,...,K$, we denote with ${\mathcal {N}}_j$ the set of instances that are present in view *j*, and with ${\mathcal {N}} = \bigcup _{j=1}^{K}\ {\mathcal {N}}_j$ the set of all the instances. Accordingly, $|{\mathcal {N}}|$ denotes the total number of instances. Every instance is associated with a class label $y_i \in {\mathcal {Y}} \subset \mathbb {N^+}$, which corresponds to a multi-class classification task, with each different integer representing a different class.

The algorithm starts by initializing the weights $w_i$ for each instance $i \in {\mathcal {N}}$, and probabilities $p_j$ for each view $j=1,2,...,K$ (Algorithm 2, line 1). Lines 3 and 4 of Algorithm 2 are the same as lines 3 and 4 of Algorithm 1 (rBoost.SH). Besides computing the predicted label, the weak classifier in irBoost.SH outputs a *class probability vector* ${\textbf{v}}_i$ for each instance (Algorithm 2, line 5), that is a confidence vector with components in [0, 1] for each class $l\in {\mathcal {Y}}$. The predicted label is used in the computation of the edge (Algorithm 2, line 7), thus indirectly also for the computation of the reward (Algorithm 2, line 8) and for the update of the shared weights (Algorithm 2, line 14). Unlike rBoost.SH, irBoost.SH uses class probabilities in the output prediction.

Because of the possibility to have incomplete views, we define the modified predicted label ${\hat{h}}^j(x_i^j)$ and the modified class probability vector $\hat{{\textbf{v}}}^j(x_i^j)$ for $i \in {\mathcal {N}}$, as follows:

1 $$\begin{aligned} {\hat{h}}^j(x_i^j) = {\left\{ \begin{array}{ll} h^j(x_i^j) &{} \text { if } i \in {\mathcal {N}}_j\\ 0 &{} \text { otherwise} \end{array}\right. } \end{aligned}$$

2 $$\begin{aligned} \hat{{\textbf{v}}}^j(x_i^j) = {\left\{ \begin{array}{ll} {\textbf{v}}^j(x_i^j) &{} \text { if } i \in {\mathcal {N}}_j\\ {0} &{} \text { otherwise} \end{array}\right. } \end{aligned}$$

Note that 0 in Eq. (1) does not correspond to a class, but it is an artificial label (note that ${\mathcal {Y}} \subset {\mathbb {N}}^+$) for unknown class label prediction for instances not contained in that view *j*. In that case, the artificial prediction, and hence unknown instance representation, will not contribute to the computation of the edge nor to the update of the weights. Accordingly, in Eq. (2), ${0}$ represents the unknown probability vector, which is, however, used in the prediction phase.

We also update the computation of the edge towards the multi-class classification task. In the original rBoost.SH, the multiplication of real and predicted labels $y_i \cdot h_t^j$ leads to 1 when they agree, i.e., $1 \cdot 1$ or $(-1) \cdot (-1)$, and to $-1$ when they disagree, i.e., $(-1) \cdot 1$ or $1 \cdot (-1)$. Analogously, we define the function ${\mathbb {I}}$, that generalizes such agreement, as follows:

3 $$\begin{aligned} {\mathbb {I}}[a = b] = {\left\{ \begin{array}{ll} 1 &{} \text {if } a=b\\ 0 &{} \text {otherwise} \end{array}\right. } \end{aligned}$$

Accordingly, we also modify the sum of multiplications of the original rBoost.SH ($\sum _{i\in {\mathcal {N}}} y_i \cdot h(x_i)$, where $y_i,\ h(x_i) \in \{-1, +1\}$) as $2 \cdot \sum _{i\in {\mathcal {N}}} \left( {\mathbb {I}}[y_i=h(x_i)] - 0.5\right)$, where ${\mathbb {I}}$ is computed through Eq. (3), and $y_i,\ h(x_i) \in {\mathbb {N}}$. More formally, we derive the updated sum of multiplications as follows:

4 $$\begin{aligned} \sum _{i\in {\mathcal {N}}} y_i h(x_i)&= \sum _{\begin{array}{c} y_i=h(x_i)\\ i\in {\mathcal {N}} \end{array}}1 - \sum _{\begin{array}{c} y_i \ne h(x_i)\\ i\in {\mathcal {N}} \end{array}}1 = \sum _{\begin{array}{c} y_i=h(x_i)\\ i\in {\mathcal {N}} \end{array}}1 - \left(n - \sum _{\begin{array}{c} y_i=h(x_i)\\ i\in {\mathcal {N}} \end{array}}1\right)\nonumber \\&= \sum _{\begin{array}{c} y_i=h(x_i)\\ i\in {\mathcal {N}} \end{array}}1 - \left(\sum _{i\in {\mathcal {N}}}1 - \sum _{\begin{array}{c} y_i=h(x_i)\\ i\in {\mathcal {N}} \end{array}}1\right) = 2\sum _{\begin{array}{c} y_i=h(x_i)\\ i\in {\mathcal {N}} \end{array}}1 - \sum _{i\in {\mathcal {N}}}1 \nonumber \\&= 2\sum _{i\in {\mathcal {N}}} {\mathbb {I}}[y_i=h(x_i)] - \sum _{i\in {\mathcal {N}}}1 = \sum _{i\in {\mathcal {N}}} 2{\mathbb {I}}[y_i=h(x_i)] - \sum _{i\in {\mathcal {N}}}1 \nonumber \\&= \sum _{i\in {\mathcal {N}}} (2{\mathbb {I}}[y_i=h(x_i)] - 1) = \sum _{i\in {\mathcal {N}}} 2({\mathbb {I}}[y_i=h(x_i)] - \frac{1}{2}) \nonumber \\&= 2 \sum _{i\in {\mathcal {N}}} \left({\mathbb {I}}[y_i=h(x_i)] - \frac{1}{2}\right) \end{aligned}$$

The above term is almost the same used in the computation of the edge (Algorithm 2, line 7), except for the fact that it is weighted. Since ${\mathbb {I}}$ compares real and predicted values, and outputs values in $\{0,1\}$, now the class label can be any positive natural number. In the case of incomplete views, the predicted and the real label for an instance that is absent in the chosen view would never be the same, because of the definition given in Eq. (1) and the assumption that class labels are from a subset of positive natural numbers. Accordingly, it will not contribute to the total sum. Formally:

$$\begin{aligned} \sum _{i\in {\mathcal {N}}} y_i{\hat{h}}(x_i) = 2 \sum _{i\in {\mathcal {N}}} ({\mathbb {I}}[y_i={\hat{h}}(x_i)] - 0.5) \end{aligned}$$

As a final remark, we want to point out that the approach followed by irBoost.SH (but also its predecessor rBoost.SH) exhibits an interesting advantage: the number of times each view is selected as the winning view can be seen as an indicator of its relevance. As emphasized in the introduction, this aspect is very useful when views correspond to different preprocessing pipelines in order to identify the most useful ones for the predictive task at hand.

**Algorithm 2:**
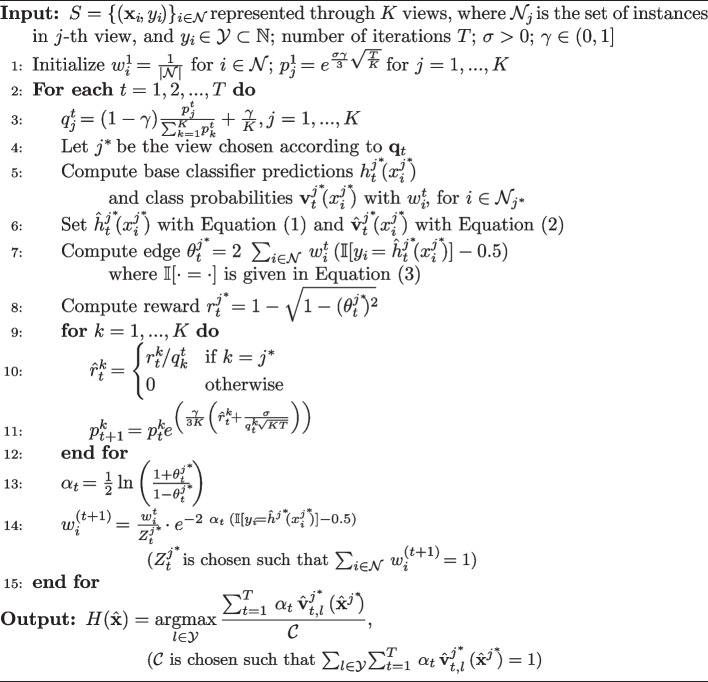
The proposed method irBoost.SH

## Experimental setting

In this section, we describe the experimental setting of the evaluation we performed to assess the effectiveness of the proposed method irBoost.SH. As introduced at the end of section ‘Introduction’, we considered two different scenarios: *i)* the classification of individuals according to 16S and/or shotgun microbiome data, which represent two, possibly incomplete, different views; *ii)* the classification of individuals considering multiple preprocessing pipelines and hyper-parameter settings to generate multiple views from the same 16S data. For the former, we consider data related to the (binary) classification of individuals in terms of presence or absence of Autism Spectrum Disorders (ASD); for the latter, we consider 16S microbiome data related to ASD and 16S microbiome data for the (multi-class) classification of individuals according to the degree (or absence, in case of healthy individuals) of the Colorectal Cancer (CRC) disease.

In the following subsections, we first describe the contexts and the datasets; then we describe the pipelines followed to define the views; finally, we describe the evaluation setting and the considered competitors.

### Autism spectrum disorder (ASD)

The Autism Spectrum Disorder (ASD) is a severe neurodevelopmental disorder that is primarily characterized by abnormal behavioral symptoms: social interaction impairment, stereotyped behavior, and restricted interests. Recent studies have shown a significant association between this disease and gut microbiome through the so-called microbiota-gut-brain axis [4, 31, 32].

The considered dataset is publicly available at the NCBI repository^1^ and consists of a cohort of 143 children with clinical diagnosis of ASD, aged 2–13 years old, and age- and sex-matched typically developing (TD) individuals (average age $5.189 \pm 0.170$; 127 males and 16 females), who attended annual physical examinations. 16S rRNA sequencing of feces samples was performed for all individuals, while shotgun metagenomic sequencing was performed only for 30 ASD and 30 TD individuals. We considered two multi-view versions of the dataset:

- **MV-ASD**, that contains pre-computed 16S rRNA and shotgun Operational Taxonomic Unit (OTU) tables as available at the Kaggle repository,^2^ where 16S data represent the first view and shotgun data represent the second view (see quantitative information in Table 1,^3^);
- **ASD-16S**, that we constructed by downloading the raw 16S rRNA sequences from NCBI and by preprocessing them using the pipelines described in ‘Data preprocessing pipelines’ section, obtaining 40 different views for all children in the study (143 ASD + 143 TD).

**Table 1:** Quantitative details of the MV-ASD dataset

	# Individuals			# Features
	ASD	TD	Total	
16S	143	111	254	1322
Shotgun	30	30	60	5619
16S Only	113	85	198	1322
Shotgun Only	0	4	4	5619
16S + Shotgun	30	26	56	6941

It is noteworthy that MV-ASD is inherently incomplete: primarily, incompleteness comes from a much lower number of individuals represented through the shotgun view with respect to those represented through the 16S view; moreover, some 16S samples were discarded during the preprocessing of the dataset (see footnote 3). Therefore, in the dataset we have individuals that are represented with (i) only 16S features, (ii) only shotgun features, or (iii) both 16S and shotgun features. Accordingly, we defined three different experimental settings, that are summarized in Table 2. The first setting (**MV-ASD-1**) considers only individuals that are represented according to (at least) the 16S view: the representation according to the shotgun view is considered only for those individuals that also have a representation in the 16S view. The second setting (**MV-ASD-2**) considers only individuals that are represented according to (at least) the shotgun view: the representation according to the 16S view is considered only for those individuals that also have a representation in the shotgun view. Finally, the third setting (**MV-ASD-3**) only focuses on those individuals who are represented according to both 16S and shotgun views. Therefore, in this setting, views are complete.

**Table 2:** Experimental settings for the MV-ASD dataset

Setting	# Individuals	Features	Classifier
MV-ASD-1 (incomplete)	254	16S	RF (SV)
	254	16S+shotgun	Concat-RF (MV)
	254	16S+shotgun	rBoost.SH (MV)
	254	16S+shotgun	irBoost.SH (MV)
MV-ASD-2 (incomplete)	60	shotgun	RF (SV)
	60	shotgun+16S	Concat-RF (MV)
	60	shotgun+16S	rBoost.SH (MV)
	60	shotgun+16S	irBoost.SH (MV)
MV-ASD-3 (complete)	56	16S+shotgun	Concat-RF (MV)
	56	16S+shotgun	rBoost.SH (MV)
	56	16S+shotgun	irBoost.SH (MV)

### Colorectal cancer (CRC)

Colorectal cancer (CRC) is the second leading cause of death among cancers in the USA [1]. Several studies suggest that gut microbiota may represent a reservoir of biomarkers that would complement existing non-invasive methods, such as the fecal immunochemical test (FIT) [1–3].

The considered dataset, hereafter called **CRC**, consists of OTU tables computed from 16S rRNA sequences for the prediction of the CRC condition between *CRC* (191 samples), *Adenoma*^4^ (241 samples) or *Control* (277 healthy samples). Sequences were downloaded and merged from three repositories^5^^,^^6^^,^^7^ selected according to the guidelines^8^ produced within ML4Microbiome COST Action CA18131,^9^ and preprocessed following the pipelines described in ‘Data preprocessing pipelines’ section, leading to a total of 40 different views.

### Data preprocessing pipelines

Amplicon sequence data preprocessing was performed using QIIME 2 [33], version 2021.2. Forward sequences were denoised with DADA2 [34], truncated at 240 bp and closed-reference clustered against SILVA database by varying the similarity threshold in $\{ 90\%, 94\%, 97\%, 99\% \}$. Each of the produced OTU tables after clustering was than subjected to feature filtering, that has been performed according to the following criteria:

- by *frequency*, i.e., on the minimum number of individuals for whom a given feature must be present, in $\{ 2, 5, 10, 50 \}$;
- by *samples*, i.e., on the minimum percentage of the individuals for whom a given feature must be present, in $\{ 2\%, 5\%, 10\%, 20\%, 50\% \}$.

Moreover, we also considered unfiltered OTU tables. In total, we obtained 40 different views, considering 4 different similarity thresholds in the clustering phase, multiplied by 10 different filtering configurations, i.e., 4 (filtering by frequency) + 5 (filtering by samples) + 1 (unfiltered).

For the ASD-16S dataset (‘Autism spectrum disorder (ASD)’ section), average reads quality scores were very good (higher than 30), except for one point in the region between 240 and 260 bp. This was the reason for truncating at 240bp in DADA2 denoising. On average, 88% of input ASD-16S sequences passed denosing and, in total 2517 of features were identified in Amplicon Sequence Variant (ASV) table, subsequently used for the clustering phase. The final number of features ranged from 219 to 1737, depending on the pipeline.

For the CRC dataset (‘Colorectal cancer (CRC)’ section), the quality of the paired-end sequences (both forward and reverse) was not particularly high (especially after 240bp). On average, around 70% of input sequences passed DADA2 denoising (trimmed at 240bp) and merging for paired-end reads. After denoising, 14055 ASV features were found, but this number was reduced by the clustering into OTUs and by the filtering step, leading to a final number of features ranging from 69 to 4402.

**Fig. 2:**
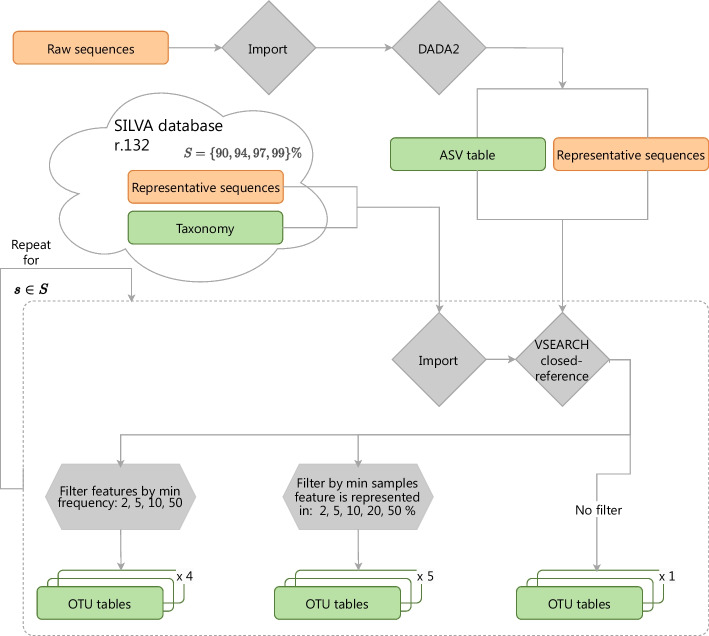
QIIME 2 data preprocessing pipeline

A complete overview of the workflow followed for sequence preprocessing is provided in the Fig. 2.

### Model evaluation and comparison

For irBoost.SH, we set the parameters $\sigma$ and $\gamma$ as suggested in [12] for rBoost.SH, namely, $\sigma =0.15$ and $\gamma =0.3$. As base classifiers we considered single decision trees (DT) and random forests (RF) with 50 trees. Since after 50 iterations the results appeared to be comparable, in the following, we only show those obtained through RF.

In order to actually assess the contribution provided by irBoost.SH in capturing the information conveyed by multiple views, we also performed the experiments with some competitor approaches. Specifically, we adopted the standard (single-view) version of RF, hereafter denoted with **RF (SV)**, for MV-ASD-1 and MV-ASD-2, as well as for all the 40 views of ASD-16S and CRC, that are considered separately. This comparison allows us to understand if the multi-view model learned by irBoost.SH outperforms all the models (2 in the case of MV-ASD-1 and MV-ASD-2, 40 in the case of ASD-16S and CRC) learned from a single view.

We also ran the experiments with the original version of rBoost.SH (hereafter denoted with **rBoost.SH (MV)**), on all the considered datasets except for the CRC dataset, on which it cannot be run due to the presence of multiple classes. Moreover, to overcome the incompleteness of the views for MV-ASD-1 and MV-ASD-2, we adopted a standard approach based on filling missing values with the mean value observed for each feature. Finally, we ran a baseline multi-view version of RF, hereafter denoted with **Concat-RF (MV)**, where the features coming from all the views are concatenated; also in this case, to manage the possible incompleteness of the views, we replaced missing values with the mean value observed for each feature.

We want to remark that, although other base learners could have been considered in our experiments (either plugged into our framework or as standalone single-view systems), they were not included since the primary focus was on the assessment of the contribution provided by the multiple available (possibly incomplete) views, rather than on the comparison of the performance achieved through different base learners.

As the evaluation strategy we adopted 10-fold cross-validation. In the case of incomplete views, the partitioning was made such that each view is split into 10 equally-sized folds. The adopted performance measures are precision, recall, accuracy, F1-score and area under the ROC curve (AUC). Note that it was not possible to collect the AUC for rBoost.SH (MV) since it does neither return a probability distribution for labels nor a score/confidence, but directly the predicted label.

## Results and discussion

**Fig. 3:**
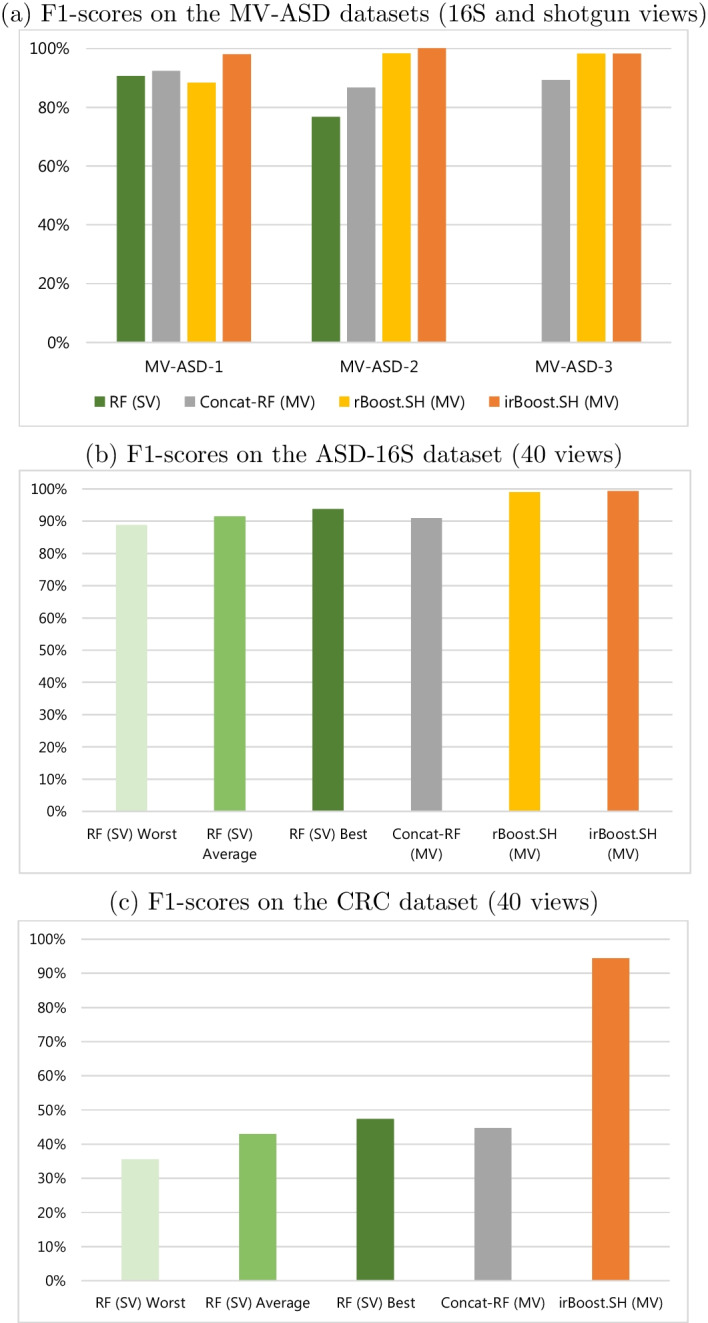
F1-scores obtained on all the analyzed datasets by irBoost.SH, and by competitor single-view (SV) and multi-view (MV) approaches

In Table 3, we show the results of the considered approaches on all the datasets in terms of all the evaluation measures, while in Fig. 3 we graphically summarize the results in terms of F1-scores. In Table 3 and Fig. 3, *RF (SV) Worst*, *RF (SV) Average* and *RF (SV) Best* denote, respectively, the worst, the average and the best result achieved among all the constructed views, by the single-view Random Forest model. The whole set of results obtained by each single view is reported in Additional file 1, while some confusion matrices are shown in Additional file 2. In the following, we mainly discuss the results in terms of F1-scores, but similar conclusions can be drawn considering the other evaluation measures.

**Table 3:** Results obtained by all the methods on all the considered datasets, in terms of Preciison, Recall, F1-score, Accuracy and AUC

	Method	Prec. (%)	Rec. (%)	F1 (%)	Acc. (%)	AUC (%)
MV-ASD-1	RF (SV)	91.31	90.35	90.70	90.94	95.67
	Concat-RF (MV)	92.78	92.05	92.34	92.52	96.27
	rBoost.SH (MV)	91.07	87.59	88.41	88.98	–
	irBoost.SH (MV)	**98.31**	**97.75**	**97.99**	**98.03**	** 99.89**
MV-ASD-2	RF (SV)	76.79	76.67	76.64	76.67	88.89
	Concat-RF (MV)	87.33	86.67	86.61	86.67	92.78
	rBoost.SH (MV)	98.39	98.33	98.33	98.33	–
	irBoost.SH (MV)	**100.00**	**100.00**	**100.00**	** 100.00**	**100.00**
MV-ASD-3	Concat-RF (MV)	89.74	89.74	89.29	89.29	94.94
	rBoost.SH (MV)	**98.39**	98.08	98.20	**98.21**	-
	irBoost.SH (MV)	98.15	**98.33**	**98.21**	**98.21**	**100.00**
ASD-16S	RF (SV) Worst	88.84	88.81	88.81	88.81	95.48
	RF (SV) Average	91.49	91.45	91.45	91.45	96.80
	RF (SV) Best	93.74	93.71	93.71	93.71	97.78
	Concat-RF (MV)	90.98	90.91	90.91	90.91	96.52
	rBoost.SH (MV)	98.97	98.95	98.95	98.95	–
	irBoost.SH (MV)	**99.31**	**99.30**	**99.30**	**99.30**	**99.99**
CRC	RF (SV) Worst	38.16	35.97	35.54	37.71	56.17
	RF (SV) Average	46.59	42.53	42.98	43.77	61.56
	RF (SV) Best	51.63	46.52	47.31	47.46	64.16
	Concat-RF (MV)	47.51	44.11	44.73	45.06	62.17
	irBoost.SH (MV)	**96.15**	**95.83**	**95.97**	**95.90**	**99.69**

The best result for each measure and dataset is highlighted in bold

Focusing on the MV-ASD datasets, we can observe that simultaneously considering both 16S and shotgun views is generally beneficial, even if they are exploited through a simple concatenation strategy, as done by Concat-RF (MV). However, in this case, the improvement obtained by adding shotgun data about 60 individuals over 254 total individuals represented through 16S data (MV-ASD-1) led to a less sensible improvement with respect to adding 16S data about 56 individuals over 60 total individuals represented through shotgun data (MV-ASD-2). The results obtained by rBoost.SH exhibit this phenomenon in a much more evident manner: on MV-ASD-1, rBoost.SH obtains worse results than RF (SV), while on MV-ASD-2 it exhibits interesting improvements. This phenomenon is possibly due to the fact that the number of missing values (replaced by the mean value of each feature) is, in the case of MV-ASD-1, much higher than in MV-ASD-2. This is supported by the fact that on MV-ASD-3, where views are complete, rBoost.SH obtains results that are comparable with those achieved by our method. This observation confirms the effectiveness of irBoost.SH in handling the incompleteness of the views.

In summary, we achieved an improvement of 8% over RF (SV), 6.1% over Concat-RF (MV), and 10.8% over rBoost.SH on MV-ASD-1; an improvement of 30.5% over RF (SV), 15.5% over Concat-RF (MV), and 1.7% over rBoost.SH on MV-ASD-2; an improvement of 10% over Concat-RF (MV) and a tie with rBoost.SH on MV-ASD-3.

The exploitation of the complementarity of the information conveyed by 16S and shotgun views, performed by irBoost.SH, is confirmed by the fact that the 16S view was selected as the winner in 51% of the iterations, while the shotgun view was selected as the winner in 49% of the iterations, emphasizing an almost equal importance and contribution of the views.

Focusing on the results on the other datasets, we can observe that the selection of the preprocessing pipeline in a single-view setting influences the results. Indeed, for the ASD-16S dataset, the F1-score ranges from about 89% (in the worst case) to about 94% (in the best case), while for the CRC dataset, it ranges from about 35% (in the worst case) to 47% (in the best case). The multi-view approach based on the concatenation, i.e., Concat-RF (MV), always performed better than the worst single-view pipeline, but was not able to reach the best single-view configuration. On the contrary, irBoost.SH provided a significant boost, with irBoost.SH reaching a F1-score of more than 99% on ASD-16S and more than 95% on CRC, achieving an improvement over Concat-RF (MV) of 9% and 114%, respectively. rBoost.SH was able to obtain good results on ASD-16S, with a disadvantage of about 0.3% over our method irBoost.SH. Like MV-ASD-3, this dataset was naturally complete, therefore, in these scenarios, the only difference with respect to irBoost.SH is the different way they combine the output. On the other hand, as already stated in ‘Model evaluation and comparison’ section, rBoost.SH cannot be run at all on the CRC dataset since it is multi-class.

In order to further confirm the significance of the obtained results, we computed the following three Wilcoxon signed-rank tests:

- **irBoost.SH** versus **RF (SV) Best**, on all the datasets except for MV-ASD-3, on which RF (SV) Best is not applicable;
- **irBoost.SH** versus **Concat-RF (MV)**, on all the datasets;
- **irBoost.SH** versus **rBoost.SH (MV)**, on all the datasets except for CRC, on which rBoost.SH (MV) cannot be run since it is multi-class.

Considering that we ran multiple tests, we corrected the obtained *p* values with the False Discovery Rate (FDR) correction proposed by Benjamini and Hochberg [35], obtaining the results reported in Table 4. As it can be observed from the table, irBoost.SH outperforms all the considered competitors at a significance level $\alpha = 0.01$.

**Table 4:** *P* values obtained by the Wilcoxon signed-rank tests, after the application of the False Discovery Rate (FDR) correction [35]

	RF (SV) Best	Concat-RF (MV)	rBoost.SH (MV)
irBoost.SH versus	<0.0001	<0.0001	0.00164

For the ASD-16S dataset, on average, each view built through a frequency-based filtering, sample-based filtering, and no filtering was selected as the winner in 2.8%, 2.3%, and 2.1% of the iterations, respectively. For the CRC dataset, on average, each view built through a frequency-based filtering, sample-based filtering, and no filtering was selected as the winner in 2.5%, 2.75%, and 2.44% of the iterations, respectively. An overview of the percentage of iterations for which each view was selected is provided in Additional file 3.

The huge improvement in terms of all the evaluation measures provided by irBoost.SH, together with the (almost) equal distribution of the times each type of views was selected, confirms that the features constructed by multiple pipelines describe the phenomenon from different, complimentary, viewpoints, that are fruitfully captured by irBoost.SH.

## Conclusion

In this paper, we proposed a novel method, called irBoost.SH, to solve multi-class classification tasks from multiple, possibly incomplete, views. Instead of discarding the information conveyed by incomplete views or filling in missing values, irBoost.SH exploits the available information of all the views, without introducing excessive approximation in the data, through a boosting process based on multi-armed bandits.

The proposed algorithm is motivated by the challenges that arise in the analysis of microbiome data, where the presence of multiple, possibly incomplete, views is very common. The obtained experimental results emphasized that the models learned by irBoost.SH were able to outperform all the models learned from each single view independently, as well as a baseline multi-view approach based on the concatenation of the features of all the views. The superiority of irBoost.SH was observed in two different tasks, i.e., in the prediction of the presence of Autism Spectrum Disorders (ASD) and in the prediction of the presence of the Colorectal Cancer (CRC) disease, where views were constructed considering 16S and shotgun data (2 views) or by applying several preprocessing pipelines (40 views). The obtained results confirmed that irBoost.SH can fruitfully be adopted for the analysis of microbiome data, to simultaneously exploit 16S and shotgun data, and to solve the issues coming from the identification of the most proper preprocessing pipeline, that in this case is fully automated.

As future work, we will adapt irBoost.SH to solve other tasks, such as regression and multi-target classification/regression. Moreover, we will investigate the possibility to make it able to work in the semi-supervised learning setting, where the class label is not available for all the training instances. Finally, we will evaluate the effectiveness of irBoost.SH in capturing the information conveyed by multiple, possibly incomplete, views also in other application domains.

### Supplementary information

**Additional file 1.** Full set of results. Description: Complete results in terms of precision, recall, F1-score, accuracy and AUROC for all the considered datasets and classifiers

**Additional file 2.** Confusion matrices. Description: Confusion matrices for all the considered datasets and classifiers

**Additional file 3.** Selected views. Description: Overview of the percentage of chosen views over iterations in irBoost.SH for all the considered datasets

## Data Availability

The datasets considered in this study for the prediction of the Autism Spectrum Disorder are available at the following links: https://www.ncbi.nlm.nih.gov/bioproject/?term=PRJNA453621 and https://www.kaggle.com/datasets/antaresnyc/human-gut-microbiome-with-asd. The datasets considered in this study for the prediction of the Colorectal Cancer Disease are available at the following links: https://www.ncbi.nlm.nih.gov/bioproject/PRJNA290926, https://www.ncbi.nlm.nih.gov/bioproject/PRJEB6070, and http://mothur.org/MicrobiomeBiomarkerCRC. All the datasets in the pre-processed form are publicly available at: https://figshare.com/collections/Processed_16S_rRNA_sequencing_multi-view_datasets/7066355. The source code of the proposed method irBoost.SH is publicly available at: https://github.com/AndreaMSBios/Multi-class-boosting-for-the-analysis-of-multiple-incomplete-views-on-microbiome-data.

## Footnotes

1 https://www.ncbi.nlm.nih.gov/bioproject/?term=PRJNA453621

2 https://www.kaggle.com/datasets/antaresnyc/human-gut-microbiome-with-asd

3 Note that the number of available TD individuals in the dataset available at the Kaggle repository is slightly less than the original dataset available at NCBI (28 missing TD individuals). This is possibly due to some preprocessing steps applied by the authors of the dataset available at the Kaggle repository.

4 A tumor that is not cancer. It starts in gland-like cells of the epithelial tissue (thin layer of tissue that covers organs, glands, and other structures within the body).

5 https://www.ncbi.nlm.nih.gov/bioproject/PRJNA290926.

6 https://www.ncbi.nlm.nih.gov/bioproject/PRJEB6070.

7 http://mothur.org/MicrobiomeBiomarkerCRC/.

8 https://doi.org/10.5281/zenodo.7382814.

9 https://www.cost.eu/actions/CA18131/.
