# Supplementary material for: Multi-class boosting for the analysis of multiple incomplete views on microbiome data
Source: BMC Bioinformatics. 2024 May 14;25:188. doi: 10.1186/s12859-024-05767-w (PMC11092168; doi:10.1186/s12859-024-05767-w)

Supplementary material Figure S2: Confusion matrices

The following confusion matrices refer to the results obtained by:

(i) RF (SV) – standard Random Forest classifier (50 estimators) applied on a single-view; for ASD-16S and CRC, we report the confusion matrices for the best model.

(ii) Concat-RF (MV) – standard Random Forest classifier applied on concatenated table of all views, after replacing missing values with the mean of each feature on datasets with incomplete views;

(iii) rBoost.SH (MV), only on datasets with binary classification, after replacing missing values with the mean of each feature on datasets with incomplete views;

(iv) the proposed model irBoost.SH (MV).

I. MV-ASD

Classes: 0 – TD, 1 - ASD

a) MV-ASD-1: RF (SV)

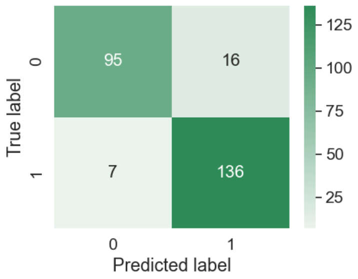

b) MV-ASD-1: Concat-RF (MV)

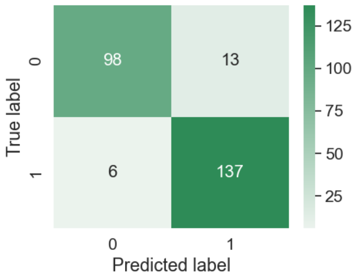

c) MV-ASD-1: rBoost.SH (MV)

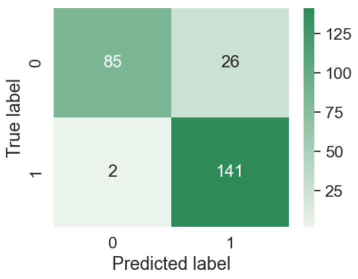

d) MV-ASD-1: irBoost.SH (MV)

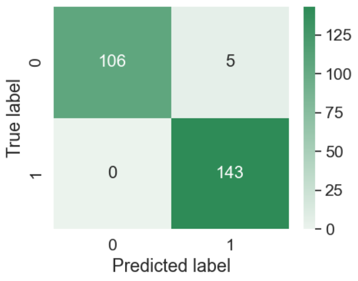

e) MV-ASD-2: RF (SV)

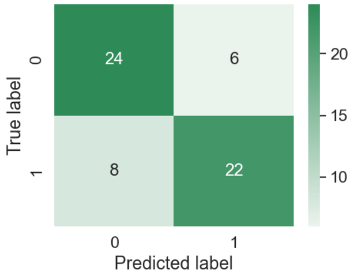

f) MV-ASD-2: Concat-RF (MV)

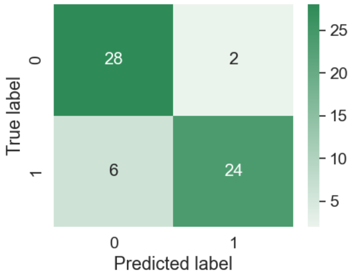

g) MV-ASD-2: rBoost.SH (MV)

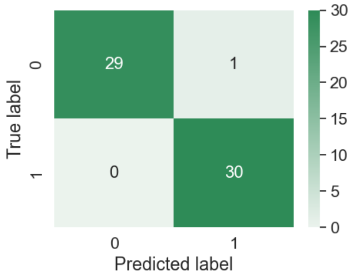

h) MV-ASD-2: irBoost.SH (MV)

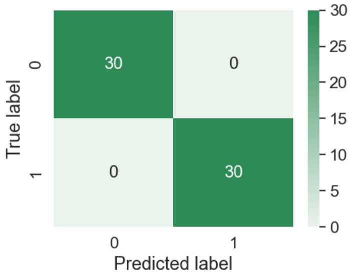

i) MV-ASD-3: Concat-RF (MV)

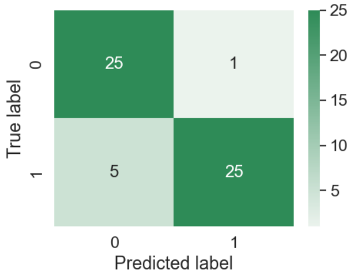

j) MV-ASD-3: rBoost.SH

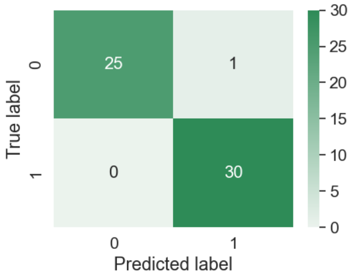

k) MV-ASD-3: irBoost.SH (MV)

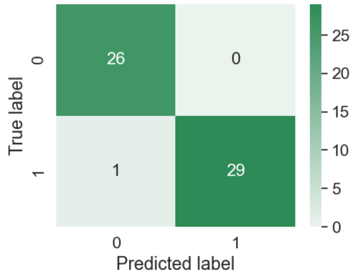

II. ASD-16S

Classes: 0 – TD, 1 - ASD

a) RF (SV) Best

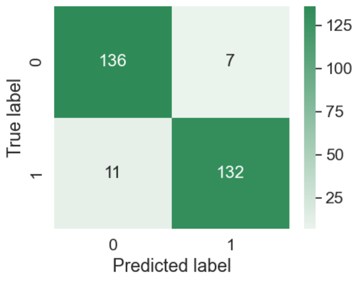

b) Concat-RF (MV)

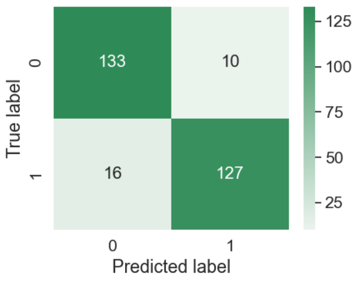

c) rBoost.SH (MV)

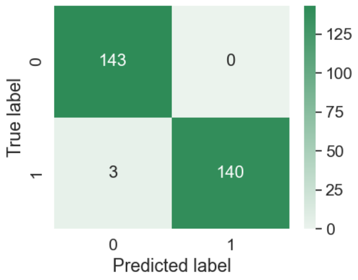

d) irBoost.SH (MV)

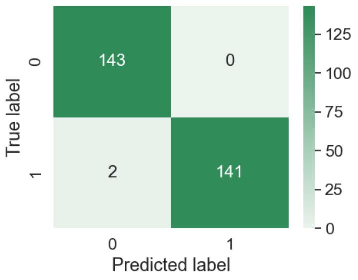

III. CRC

Classes: 0 – Healthy, 1 – Adenoma, 2 - CRC

a) RF (SV) Best

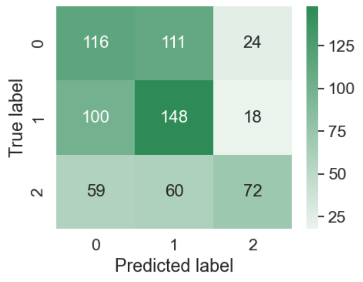

b) Concat-RF (MV)

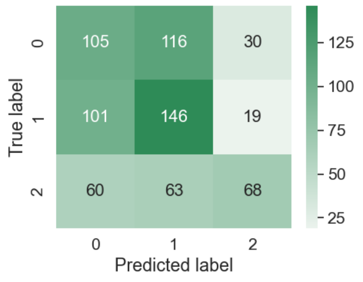

c) irBoost.SH (MV)

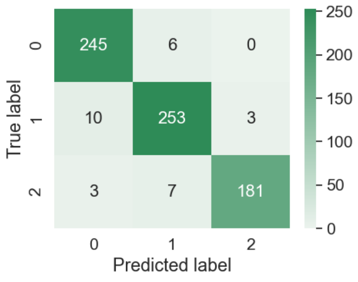

Supplement: Supplementary file 2 — Additional file 2. Confusion matrices. Description: Confusion matrices for all the considered datasets and classifiers [file 12859_2024_5767_MOESM2_ESM.pdf]
